# Heat treatment in the presence of arginine increases the emulsifying properties of soy proteins

**DOI:** 10.1016/j.fochx.2023.100567

**Published:** 2023-01-07

**Authors:** Hiroshi Kano, Kentaro Shiraki

**Affiliations:** a Research Institute for Creating the Future, Fuji Oil Holdings INC, 1 Sumiyoshi-cho, Izumisano-shi, Osaka 598-8540, Japan; b Faculty of Pure and Applied Sciences, University of Tsukuba, 1-1-1 Tennodai, Tsukuba, Ibaraki 305-8573, Japan

**Keywords:** Commercial soy protein isolate, Arginine, Aggregation, Flocculation, Emulsifying properties

## Abstract

This study aimed to improve the emulsifying properties of commercial soy protein isolates (CSPIs). CSPIs were thermally denatured without additives (CSPI_H) and with arginine (CSPI_A), urea (CSPI_U), and guanidine hydrochloride (CSPI_G), which improve protein solubility to prevent aggregation. These additives were removed by dialysis, and the samples were lyophilized. CSPI_A resulted in high emulsifying properties. FT-IR analysis showed that the β-sheet content in CSPI_A was reduced compared to that of untreated CSPI (CSPI_F). Fluorescence analysis showed that the tryptophan-derived emission peak of CSPI_A shifted between CSPI_F and CSPI_H which was exposed to hydrophobic amino acid chains with aggregation. As a result, the structure of CSPI_A became moderately unfolded and exposed the hydrophobic amino acid chains without aggregation. The CSPI_A solution had a more reduced oil–water interface tension than other CSPIs. These results support that CSPI_A attaches efficiently to the oil–water interface and produces small, less flocculated emulsions.

## Highlights

• Heat treatment with arginine caused thermal protein denaturation without aggregation.
• The protein structure was still denatured after the removal of arginine.
• The protein remained moderately exposed in the hydrophobic part.
• The protein produced small, less flocculated emulsions compared to untreated.

## Introduction

Sodium caseinate is widely used in the food industry as a natural emulsifier (Dickinson, 1994, Dickinson, 1999, Dickinson, 2001, Dickinson, 2010) for clean-label products. The demand for plant-based emulsifiers is increasing, not only from a health perspective but also from that of sustainability and ethical considerations. However, plant-based emulsifiers are currently under development (McClements & Gumus, 2016) due to their insufficient emulsifying properties. This study was aimed to improve the emulsification properties of commercial soy protein isolates (CSPIs). Soybean is produced worldwide and is a nutritious food with a high protein content. CSPI is widely used in the food industry to add nutrient value and gelling properties to foods.

Many studies aimed to improve the emulsifying properties of soy protein isolate (SPI) (Nishinari et al., 2014, Nishinari et al., 2018). Previous studies can be divided into three main approaches. The first approach is to recover only the fractions with high emulsifying properties from SPI (Sirison et al., 2021). The second approach is to modify SPIs with enzymes. These enzymes include protease (Jung et al., 2005, Kim et al., 1990, Qi et al., 1997), phosphatase (Campbell, Shih, & Marshall, 1992), and protein-glutaminase (Suppavorasatit, Mejia, and Cadwallader, 2011). The third approach is to denature SPIs. This approach can be further divided into heat treatment (Keerati-u-rai and Corredig, 2009, Lakemond et al., 2000, Li et al., 2020, Liu and Tang, 2013, Luo et al., 2013, Palazolo et al., 2004, Wang et al., 2019) and the use of denaturants (Nir, Feldman, Aserin & Grati, 1994).

For use in the food industry, the first approach is not preferable because it is a complex process that produces unwanted by-products. The second approach, especially proteolysis by enzymes, is used commercially, but its use in food products is regulated differently in different countries. In the third approach, heat treatment reduces the solubility of the proteins themselves in water due to the aggregation that occurs between the proteins (Palazolo et al., 2004, Lakemond et al., 2000). Denaturation with chemical reagents is not suitable for food applications, and this effect is reported to be concentration-dependent and reversible (Nir et al., 1994).

This study aimed to unfold CSPIs by heat treatment with additives to prevent aggregation. Ren et al. (2018) reported that the heat-induced aggregation of proteins is caused by interactions between hydrophobic amino acid residues exposed on the surface because of unfolding. Therefore, aggregation could be suppressed by adding a substance to the protein solution that inhibits these hydrophobic interactions. Urea and guanidine hydrochloride are well-known inhibitors of hydrophobic interactions. Arginine has also been reported to inhibit protein aggregation during heating (Shiraki et al., 2015, Takai et al., 2013, Hong et al., 2017, Inoue et al., 2014, Miyatake et al., 2016, Jahan and Nayeem, 2018). Some reports suggest that the addition of arginine itself improves protein emulsification (Li et al., 2020, Guo et al., 2021, Cao et al., 2022). The purpose of using additives in this study was to reduce protein aggregation during heating, and the additives were removed by dialysis.

## Materials and methods

### Materials

CSPI (Fujipro-F), canola oil, medium-chain triglyceride (MCT, product name MCT64) oil, and rapeseed oil were supplied from Fuji Oil (Osaka, Japan). Fujipro-F is a standard commercial SPI that is untreated with any enzymatic or chemical modifications. Fujipro-F was heat-treated to 140 °C for 10 s for sterilization and powdered by spray-drying (Kawaguchi, Kita, Shinyashiki, Yagihara, & Fukuzaki, 2018). Deuterium oxide and Rhodamine B were purchased from Sigma-Aldrich (St. Louis, MO, US); BODIPY 500/510 C4 C9 was purchased from Thermo Fisher Scientific (Waltham, MA, US); Seaprep Agarose was purchased from Lonza (Basel, Switzerland); dialysis tubes (MWCO 3500 g/mol) were purchased from Nihon Medical Science (Osaka, Japan); and all other reagents were purchased from Kishida Chem (Osaka, Japan).

### Sample preparation

**Fig. 1:**
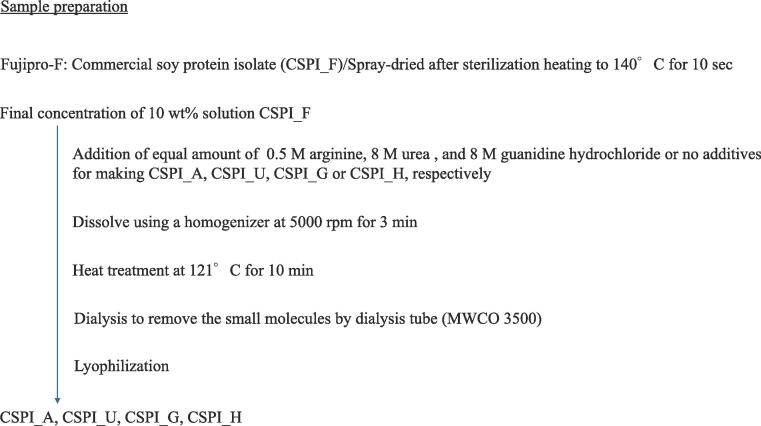
Sample preparation.

Fig. 1 shows the sample preparation.

**Sample preparation of CSPI_F**: 200 g Fujipro-F was dissolved in 1800 g of distilled water at 5000 rpm for 3 min using a homogenizer (MARK II Model 2.5, Primix, Hyogo, Japan). The obtained sample was named ‘CSPI_F.’.

**Sample preparation of CSPI_A**: 21.8 g arginine (equivalent to 0.25 M in 500 mL) was added to 300 g 10 wt% CSPI_F. Then, the arginine and CSPI_F mixture was well stirred by a homogenizer at 5000 rpm for 3 min. 10 wt% CSPI_F was then added to the arginine and CSPI_F mixture for a total volume of 500 mL. The mixture of 50 g was placed in a 100-mL heat-resistant pressure bottle, and then the bottle was heated to 121 °C for 10 min by an autoclave (HICLAVE HG-50, Kitahama Manufacturing, Osaka, Japan). The bottles were cooled under running water for 30 min after heating. The mixture was placed in a MWCO 3500 g/ L dialysis tube. Dialysis was performed three times to remove ions and small molecules. The final electrical conductivity of the distilled water side was < 0.1 mS/cm. The obtained sample was named ‘CSPI_A.’.

**Sample preparation of CSPI_U**: 120.1 g urea (equivalent to 4.0 M in 500 mL) was added to 300 g of 10 wt% CSPI_F. 10 wt% CSPI_F was then added to the urea and CSPI_F mixture for a total volume of 500 mL. Then, the sample was heated in the same manner as the preparation of CSPI_A. After that, the heated sample was subjected to dialysis, and the obtained sample was named ‘CSPI_U.’.

**Sample preparation of CSPI_G**: 191.1 g guanidine hydrochrolide (equivalent to 4.0 M in 500 mL) was added to 300 g of 10 wt% CSPI_F. 10 wt% CSPI_F was then added to the guanidine hydrochrolide and CSPI_F mixture for a total volume of 500 mL. Then, the sample was heated in the same manner as the preparation of CSPI_A. Afterward, the heated sample was subjected to dialysis, and the obtained sample was named ‘CSPI_G.’.

**Sample preparation of CSPI_H**: 50.0 g of 10 wt% CSPI_F was heated in the same manner as the preparation of CSPI_A. Then, the heated sample was subjected to dialysis, and the obtained sample was named ‘CSPI_H.’.

### Emulsion properties

#### Emulsion preparation

Emulsion preparation was performed as described previously by Amine, Dreher, Helgason, & Tadros. (2014) with modifications to the composition and preparation step of the emulsions. Briefly, the aqueous phase was a 100 mM phosphate buffer solution prepared at pH 7, containing 0.05, 0.1, 0.5, and 1.0 wt% sample in the emulsion. This experiment used medium-chain triglycerides (MCT) because MCT oil does not crystalize at 4 °C and has a less influence on the emulsification caused by changes in the state of the fats and oils. The emulsion containing 80 wt% aqueous phase and 20 wt% oil phase was first premixed for 30 *sec* using a vortex mixer at room temperature and then emulsified amplitude 60 % for 30 s using an ultrasonic homogenizer (Sonifier SFX150, Emerson Electric, MO, US) in an ice water bath. The sample volume was 5 g in a test tube. The diameter of the probe was 3.2 mm. The intensity was set to 150 W. The premixing and emulsification steps were repeated twice to obtain the emulsion. The emulsions were stored at 4 °C before use.

#### Emulsion microstructure

Emulsions containing 1 wt% sample and 20 wt% MCT were observed under a fluorescence microscope (BZ-X800, Keyence, Osaka, Japan). The resolution of the camera was 12.5 megapixels and the level of magnification was ×1000. The oil and protein were stained with BODIPY 500/510 C4 C9 (Thermo Fisher Scientific) and Rhodamine B (Sigma-Aldrich), respectively. 50 µL of emulsion solution, 50 µL of solution containing 500 µg/mL of BODIPY, and 200 µg/mL of Rhodamine B were mixed by pipetting. The mixture was named mixture A. Then, 10 µL of mixture A and 90 µL of 2 wt% heated SeaprepTM Agarose (Lonza, Basel, Switzerland) solution were mixed gently in a 0.5 mL plastic tube. This mixture was named mixture B. 50 µL of mixture B was dropped onto a glass slide.The final mixture B was covered with a coverslip. The slides were stored in a dark room at 10 °C overnight before observation.

#### Emulsion size distribution

The emulsions were measured after 1 and 7 days of storage at 4 °C. The emulsion size distribution was analyzed as described previously by Koo et al. (2019) using a laser diffraction particle size analyzer (SALD 2300, Shimadzu, Kyoto, Japan). To check for flocculation between emulsion particles and the emulsifying properties (Liu & Tang, 2013), the emulsions were diluted with distilled water or 1 wt% sodium dodecyl sulfate (SDS) solution to achieve an absorbance of 0.7–1.2 and measured at 25 °C. The refractive index was set to 1.45–0.50i.

### Interface tension measurement

The interface tension of the oil-in-water sample was measured by an automated drop tensiometer (DropMaster, DMs-401, Kyowa Interface Science, Saitama, Japan). The supplied software (FEMAS) was used for analysis. The measurement was performed according to the method reported by Sirison et al., 2021, Ishii et al., 2017. The aqueous phase was a 100 mM phosphate buffer solution prepared at pH 7 containing 0.001, 0.01, and 0.1 wt% sample. The oil phase was refined commercial rapeseed oil. An oil droplet was formed by placing a hook-shaped syringe into aqueous solutions within a cuvette. The temperature of the aqueous phase was controlled at 20 °C ± 1 °C with a water circulation system connected to a thermostat. The interface tension was calculated using the Young–Laplace formula from the droplet shape 350 s after droplet formation. The measurements were repeated three times per point, with three measurements per test section. The interface tension of the oil and 100 mM phosphate buffer without CSPI was 25.2 ± 0.5 mN/m.

### Secondary structure analysis

The samples were dissolved or dispersed in D_2_O to 5 wt% and measured using Fourier transform infrared spectroscopy (FT-IR) (Nicolet iZ10, Thermo Fisher Scientific, Waltham, MA, USA). The supplied software (OMNIC) was used for analysis. The procedure for the spectroscopic analysis was based on the method described by Sakuno et al., 2008, Sakuno et al., 2008. For each spectrum, 128 scans were carried out at 2 cm^−1^ resolution by the single reflection ATR method. In the software (OMNIC), advanced ATR correction was applied to the peak, and the secondary structure-derived peaks were identified after peak decoupling of the amide Ⅰ region (1600–1700 cm^−1^) following the method described by Susi & Byler (1986). Peak detection by a second derivative (sensitivity, low; full width at half maximum, 4) and peak separation by curve fitting (noise target value, 1; baseline, none) was performed on the absorption spectrum of the amide I region. Gauss/Lorenzian (aqueous solution) was used as the fitting function. Peak positions were fixed, and the upper limit of the half-width was set to 20 cm^−1^ to avoid irregular fitting. The sum of the separation peak areas identified between 1700 and 1600 cm^−1^ was defined as 100 %. The content of α-helix to β-sheet structures was determined from the origin of the identified peaks.

### Fluorescence spectroscopy

The samples were dissolved or dispersed in ultra-pure water to 0.02 wt% and measured using a fluorospectrometer (Duetta, Horiba, Kyoto, Japan). The supplied software (EzSpec) was used for analysis. Sample solutions were placed into quartz cuvettes with an optical path length of 1 cm. Measurements were taken with stirring at 1000 rpm. The excitation wavelength was set at 280 nm for tryptophan quenching. Emission spectra were recorded in the range between 300 and 400 nm. Excitation and emission slits were set at 5 nm. All spectra were the average of at least three repeated scans.

### Statistical analysis

All measurements were repeated at least three times. The obtained values represent the mean ± standard errors. Significant differences were considered *p*-values of < 0.05 with Tukey’s significant difference test. Analyses were performed using the SPSS software (IBM SPSS Statistics 22, Armonk, NY, USA).

## Results and discussion

### Emulsion properties

#### Appearance

Emulsified samples were prepared as follows: The aqueous phase of 80 wt% was 100 mM Na-phosphate buffer (pH 7), containing 0.05, 0.1, 0.5, and 1.0 wt% samples in the emulsion. The oil phase of 20 wt% was MCT. These samples were emulsified by ultrasonic homogenization.

**Fig. 2:**
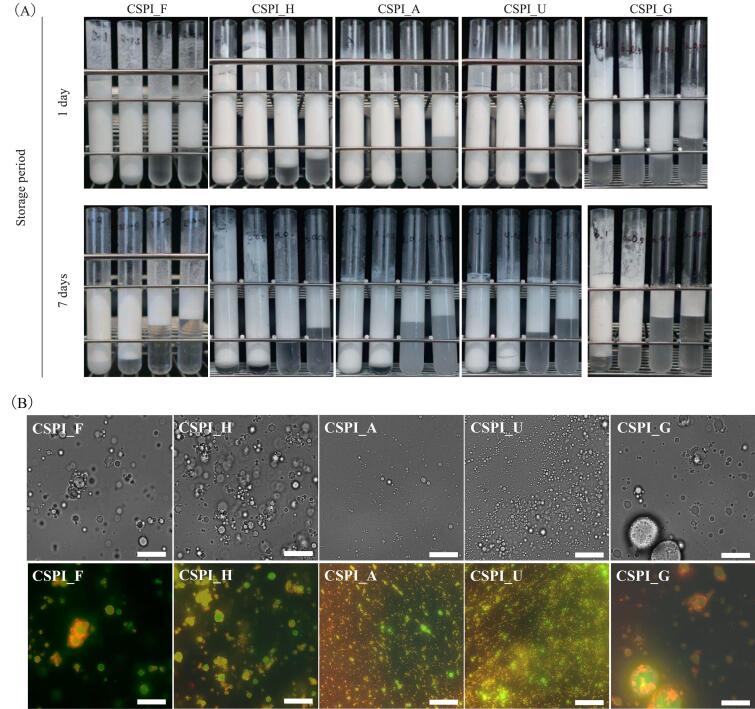
(A) The appearance of the CSPI emulsions.Sample concentration in the emulsions from left to right: 1, 0.5, 0.1, and 0.05 wt%. These samples were stored at 4 °C for 1 or 7 days. (B) Microscopic CSPI emulsion images. The emulsion contained a 1 wt% sample and 20 wt% MCT. The upper row is optical microscopic images. The lower row is fluorescent microscopy images. Oil and proteins are stained green and red, respectively. Scale bars = 20 μm. (For interpretation of the references to colour in this figure legend, the reader is referred to the web version of this article.)

Fig. 2A shows the appearance of the emulsions. All low-sample concentration emulsions (0.05 and 0.1 wt%) showed aqueous phase separation after one week of storage at 4 °C. In contrast, the high-concentration protein samples were different from the low-concentration samples. CSPI_A and CSPI_U at 0.5 wt% protein concentration did not show separation at 1-day storage, and these samples showed higher emulsification stability compared to other samples at the same concentration. Interestingly, 0.1 wt% and 0.05 wt% CSPI_A showed cloudiness in the aqueous phase, implying the presence of a fine emulsion. Taken together, supplementing the CSPI heat treatment with arginine or urea helped to improve the emulsifying properties even after the additive was removed by dialysis. CSPI_F, CSPI_H, and CSPI_G formed multiple aggregates on the walls of the test tubes. Emulsions with CSPI_H showed similar stability to emulsions with CSPI_A and U. The result of this was thickening due to protein aggregation, as Palazolo et al., 2004, Lakemond et al., 2000, Wang et al., 2019 reported.

#### Emulsion microstructure

The sample emulsions containing 1 wt% CSPIs and 20 wt% MCT were observed by optical and fluorescent microscopy. Oil and proteins were stained with BODIPY (green) and Rhodamine B (red), respectively. The aggregates observed at the top of the test tubes of CSPI_H and CSPI_G that stored for 7 days (Fig. 2A) are consistent with the microscopic images (Fig. 2B). Optical microscopy showed a high number of aggregates in CSPI_F, CSPI_H, and CSPI_G (Fig. 2B). CSPI_G formed the biggest aggregates. Fluorescence microscopy showed that the oil droplets in the CSPI_F, CSPI_H, and CSPI_G samples were surrounded by protein aggregates. CSPI_G formed large protein aggregates. In contrast, the CSPI_A and CSPI_U samples formed fine emulsions without aggregates.

#### Droplet size distribution

**Fig. 3:**
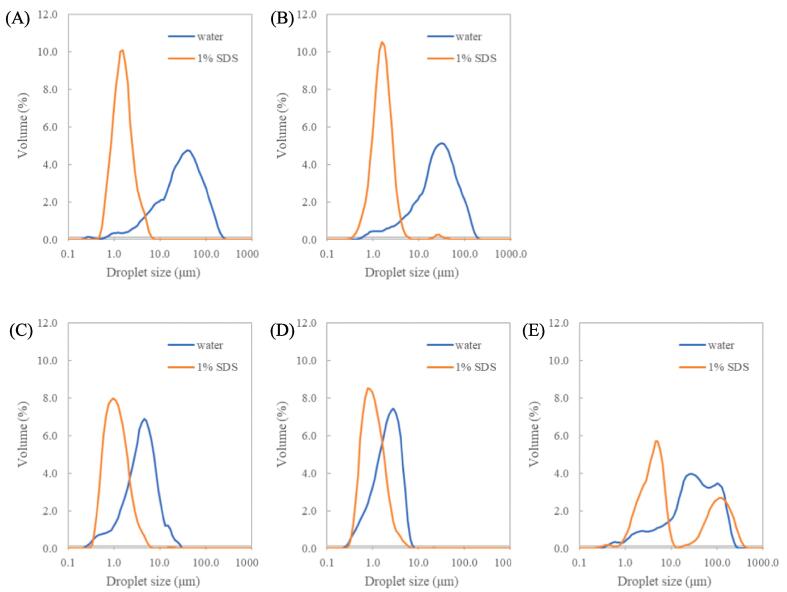
Emulsion size distribution. Emulsions were measured after 1 day of storage at 4 °C. The emulsions contained a 1 wt% sample and 20 wt% MCT diluted with water or 1 % SDS. (A) CSPI_F, (B) CSPI_H, (C) CSPI_A, (D) CSPI_U, and (E) CSPI_G.

The size distribution of the emulsion droplet containing 1 wt% CSPIs and 20 wt% MCT was measured using a laser diffraction particle size analyzer (Fig. 3). The samples were diluted with water to obtain the appropriate absorbance. The emulsions diluted with 1 wt% SDS were used as control. CSPI_F and CSPI_H diluted with water and SDS were significantly different in terms of droplet size, suggesting the presence of flocculated emulsions. The flocculation of emulsions might reduce the flowability of the emulsions and lead to thickening, inhibiting water and oil phase separation as shown in Fig. 2A, as reported by Palazolo (2004), Lakemond et al., 2000, Wang et al., 2019.

While the CSP_A and CSPI_U distilled water dilution samples also had larger droplet sizes than the SDS dilution samples, the difference was smaller than the other samples and there was less flocculation between emulsions. These results are consistent with the results in Fig. 2B. In contrast, the emulsion with CSPI_G showed two separate peaks in the presence of water and a larger droplet size even in the presence of SDS, suggesting that CSPI_G had weak emulsifying properties and was highly aggregated even in presence of SDS. The diameter of CSPI_G was clearly larger than that of CSPI_F and CSPI_H, indicating that CSPI_G was difficult to emulsify. On the other hand, CSPI_A showed a small diameter with a normal distribution; hence, CSPI_A was presumed to be easily emulsified by simple treatments such as sonication.

The results of emulsion particle size measurements after storage in a refrigerator for 1 or 7 days and dilution with distilled water are shown in Table 1. A comparison of the average diameter size of the emulsions after 1 day of storage showed that the emulsions of CSPI_A and CSPI_U had smaller droplet sizes compared with other emulsions of the same concentration (Table 1). These data were supported by the results of optical and fluorescence microscopy as shown in Fig. 2B. The emulsions remained stable even after 7 days of storage. The droplet size of CSPI_A and CSPI_U were one order of magnitude smaller than CSPI_H. On the other hand, the emulsions with CSPI_G were larger, and the emulsion size increased with time below 0.5 wt%.

**Table 1:** Volume-weighted average diameters diluted by distilled water.

Samples		Diameter of droplets (μm)															
		Sample concentration (wt%)															
		1				0.5				0.1				0.05			
After 1 day of storage																	
CSPI_F		26.7	±	1.8	^a^	38.3	±	5.8	^a^	79.4	±	30.8	^a^	106.5	±	59.1	^a^
CSPI_H		16.5	±	1.8	^b^	20.9	±	1.7	^b^	23.3	±	2.8	^a^	30.6	±	4.0	^a^
CSPI_A		3.0	±	0.3	^c^	6.7	±	1.5	^bc^	10.9	±	2.3	^a^	15.1	±	1.8	^a^
CSPI_U		2.0	±	0.3	^c^	3.2	±	0.3	^c^	8.5	±	1.5	^a^	14.9	±	2.6	^a^
CSPI_G		31.6	±	2.7	^a^	40.3	±	1.6	^a^	72.9	±	15.8	^a^	1395.6	±	553.3	^a^
																	
After 7 days of storage																	
CSPI_F		24.6	±	1.5	^a^	32.6	±	3.2	^a^	85.8	±	46.5	^a^	107.5	±	54.4	^a^
CSPI_H		13.7	±	1.5	^b^	19.4	±	1.2	^b^	25.2	±	1.1	^a^	27.6	±	3.8	^ab^
CSPI_A		2.9	±	0.1	^c^	5.4	±	0.6	^c^	8.8	±	1.2	^a^	14.9	±	0.4	^ab^
CSPI_U		1.9	±	0.2	^c^	3.4	±	0.3	^c^	7.7	±	1.5	^a^	12.4	±	1.8	^ab^
CSPI_G		28.0	±	2.6	^a^	47.2	±	2.6	^d^	89.6	±	26.5	^a^	205.0	±	15.1	^ac^

The data are expressed as the means ± standard errors. The results labeled with the same letters within a column are not significantly different (*p* < 0.05).

### Measurement of interfacial tension of oil-in-water samples

Table 2 lists the interfacial tension of oil-in-water CSPI samples. The aqueous phase was a 100 mM phosphate buffer solution prepared at pH 7 and the oil phase was commercial rapeseed oil. The measurements were carried out at 20 °C. CSPI_G could not be measured due to its significantly low dissolubility. At a sample concentration of 0.001 wt%, the interfacial tension was close to that of the blank (100 mM phosphate buffer). At 0.01 wt%, the differences between the samples were significant, with CSPI_H showing a lower interfacial tension than that of CSPI_F and even lower interfacial tension for CSPI_A and CSPI_U. The large standard errors of CSPI_F and CSPI_H are probably due to the aggregation of the proteins and the insufficient amount of protein that could be adsorbed at the oil–water interface. On the other hand, CSPI_A and CSPI_U had a high adsorption efficiency at the oil–water interface due to the lack of protein aggregation, and even 0.01 wt% could surround the oil droplet, so it is reasonable that the value of the interface tension was highly reproducible. The results in Table 2 also suggest that CSPI_A and CSPI_U could be oriented efficiently at the oil–water interface with less protein at the same concentration compared to the other samples. Therefore, fine emulsions could be maintained even at lower sample concentrations, as shown in Table 1.

**Table 2:** Interfacial tension, secondary structure, and fluorescent spectroscopy of sample solutions.

Samples	Interfacial tension (mN/m)												Secondary structure (%)								Fluorescence Spectroscopy			
	Protein concentration (wt%)												β-sheet				α-helix				λ^max^ (nm)			
	0.1				0.01				0.001															
CSPI_F	14.4	±	0.3	^a^	21.4	±	0.7	^a^	25.2	±	0.4	^a^	39.9	±	0.9	^a^	8.5	±	1.3	^a^	328.1	±	1.3	^a^
CSPI_H	13.8	±	0.4	^ab^	17.6	±	1.2	^b^	24.7	±	0.2	^ab^	39.9	±	0.4	^a^	11.3	±	1.3	^a^	344.6	±	0.8	^b^
CSPI_A	12.5	±	0.1	^c^	14.5	±	0.1	^c^	24.4	±	0.2	^b^	27.6	±	0.9	^b^	4.5	±	2.1	^a^	341.2	±	0.6	^c^
CSPI_U	13.2	±	0.3	^bc^	15.4	±	0.0	^bc^	24.2	±	0.1	^b^	21.7	±	1.0	^c^	9.9	±	3.5	^a^	339.1	±	0.6	^c^
CSPI_G	N/A				N/A				N/A				30.0	±	0.4	^b^	16.5	±	3.0	^a^	346.2	±	0.9	^b^

The results labeled with the same letters within a column are not significantly different (p < 0.05).

### Secondary structure analysis

**Fig. 4:**
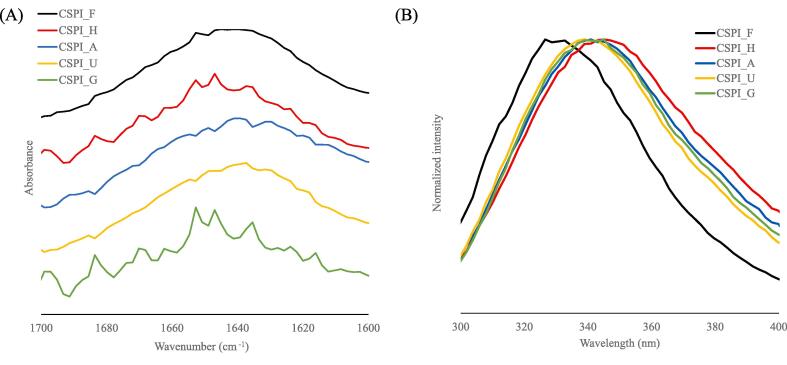
(A) FT-IR spectrum of 5 wt% CSPIs in D_2_O. The spectra were subtracted by the peak derived from D_2_O and were normalized. (B) The fluorescence emission spectrum of 0.02 wt% CSPIs in distilled water. The excitation wavelength was 280 nm.

To investigate conformational changes in CSPI due to heat treatment and additives, FT-IR measurements were carried out (Fig. 4 (A)). CSPI_F had a characteristic peak at 1653 cm^−1^, which was also present in CSPI_H and CSPI_G. CSPI_A and CSPI_U had an overall broad waveform, while CSPI_H and CSPI_G had a jagged waveform.

Peak detection of a second derivative and peak separation by curve fitting was performed at the absorption spectrum of the amide I region. The sum of the separation peak areas identified between 1700 and 1600 cm^−1^ was defined as 100 %. The content of α-helix to β-sheet structures was determined from the origin of the identified peaks (Table 2). The β-sheet structure content of CSPI_H was similar to that of CSPI_F, potentially simply because Fujipro-F was heated for sterilization purposes. The β-sheet content in CSPI_F and CSPI_H was close to the value reported by Hu et al. (2013) as the SPI solution was intensely ultrasonicated. CSPI_A, CSPI_U, and CSPI_G, which were heated with additives, all had a lower amount of β-sheets than CSPI_F. An increased β-sheet content caused protein aggregation through hydrophobic interactions between molecules (Lee, Lefevere, Subirade, & Paquin, 2007). This result was consistent with that of the emulsions with CSPI_A and CSPI_U, which had less flocculation (Fig. 2B). On the other hand, the FT-IR results for CSPI_G were inconsistent with the results in Fig. 2B, suggesting the presence of other factors.

### Fluorescence spectroscopy

Kishore, Kundu, & Kayastha (2012) reported that a redshift is caused by the fact that tryptophan residues are replaced from the less polar interior of the protein to solvent-exposed regions upon unfolding. CSPI_G had the largest redshift, suggesting that tryptophan is most exposed on the protein surface (Table 2). The FT-IR results could not explain the aggregation properties of CSPI_G, but the present results suggest that the exposure of the hydrophobic amino acid tryptophan to the protein surface led to hydrophobic interactions between the proteins, leading to aggregation. CSPI_H also showed a large redshift, and heat treatment without additives also unfolded and exposed hydrophobic surfaces (Ren et al., 2018). CSPI_A and CSP_U were redshifted compared to CSPI_F, but to a less extent than CSPI_H and G. This means that tryptophan residues were not so exposed that hydrophobic interactions between protein molecules caused aggregation, but they were moderately exposed and unfolded.

From these results, CSPI_A and U were inferred to have a moderately unfolded terminal structure, and exposure of the hydrophobic amino acid residues was attributed to an increased affinity for oil, which was more readily adsorbed at the oil–water interface, and less-aggregated protein was efficiently arranged at the oil–water interface.

Guanidine hydrochloride has a strong unfolding effect due to the weakening hydrophobic and electrostatic interactions. Thus, aggregation occurred due to the interaction of hydrophobic parts exposed by chemical denaturation after the removal of guanidine hydrochloride by dialysis. On the other hand, the unfolding effect of urea on proteins is weaker than that of guanidine hydrochloride. Similarly, arginine is thought to act mainly on the aromatic ring; hence, arginine itself has no denaturing effect (Shiraki et al., 2015).

## Conclusions

This study demonstrated that the addition of arginine or denaturing agents inhibited protein aggregation during heat denaturation and improved protein emulsification. Arginine and urea behaved as hypothesized. However, guanidine hydrochloride induced severe aggregation after its removal, and its emulsifying properties were weak. Not only thermal denaturation but also irreversible denaturation by guanidine hydrochloride occurred.

Suggested mechanisms for improving the emulsification of proteins are as follows: 1) Heating protein solutions with arginine or urea moderately unfolds the protein structure and exposes hydrophobic amino acid residues even after removing these additives, 2) the moderately exposed hydrophobic amino acid residues facilitate adsorption to the oil–water interface, and little-aggregated proteins allow smaller quantities to surround oil droplets, and 3) the proteins prepared in this study suppressed aggregation between proteins, which also reduced flocculation between emulsions, enabling the production of fine emulsions.

The method used in this study is a very versatile and safe for improving protein emulsification as it only requires the addition of arginine and heating. Herein, the arginine was removed, but it is a type of amino acid that is unlikely to be harmful to the human body and could easily be produced commercially without removal. This method could also be applied to isolated proteins such as peas and chickpeas with globulins as well as SPI.

## CRediT authorship contribution statement

**Hiroshi Kano:** Writing – original draft, Conceptualization, Methodology, Investigation. **Kentaro Shiraki:** Writing – review & editing, Supervision.

## Declaration of Competing Interest

The authors declare that they have no known competing financial interests or personal relationships that could have appeared to influence the work reported in this paper.

## Data Availability

Data will be made available on request.
